# Myocardial protection from ischemia/reperfusion injury by exogenous galanin fragment

**DOI:** 10.18632/oncotarget.15071

**Published:** 2017-02-03

**Authors:** Andrei Timotin, Oleg Pisarenko, Maria Sidorova, Irina Studneva, Valentin Shulzhenko, Marina Palkeeva, Larisa Serebryakova, Aleksander Molokoedov, Oksana Veselova, Mathieu Cinato, Helene Tronchere, Frederic Boal, Oksana Kunduzova

**Affiliations:** ^1^ National Institute of Health and Medical Research (INSERM), Toulouse, France; ^2^ University of Toulouse, UPS, Institute of Metabolic and Cardiovascular Diseases, Toulouse, France; ^3^ Russian Cardiology Research-and-Production Complex, Moscow, Russian Federation, Russia

**Keywords:** galanin (2-11), heart, ischemia and reperfusion, energy metabolism, ROS production

## Abstract

**Background and purpose:**

Galanin is a multifunctional neuropeptide with pleiotropic roles. The present study was designed to evaluate the potential effects of galanin (2-11) (G1) on functional and metabolic abnormalities in response to myocardial ischemia-reperfusion (I/R) injury.

**Experimental approach:**

Peptide G1 was synthesized by the 9-fluorenylmethoxycarbonyl (Fmoc)-based solid-phase method. The chemical structure was identified by ^1^H-NMR spectroscopy and mass spectrometry. Experiments were conducted using a rat model of I/R injury *in vivo*, isolated perfused rat hearts *ex vivo* and cultured rat cardiomyoblast H9C2 cells *in vitro*. Cardiac function, infarct size, myocardial energy metabolism, hemodynamic parameters, plasma levels of creatine kinase-MB (CK-MB) and lactate dehydrogenase (LDH) were measured in order to evaluate the effects of G1 on myocardial I/R injury.

**Key results:**

Treatment with G1 increased cell viability in a dose-dependent manner, inhibited cell apoptosis and excessive mitochondrial reactive oxygen species (ROS) production in response to oxidative stress in H9C2 cells. Pre- or postischemic infusion of G1 enhanced functional and metabolic recovery during reperfusion of the ischemic isolated rat heart. Administration of G1 at the onset of reperfusion significantly reduced infarct size and plasma levels of CK-MB and LDH in rats subjected to myocardial I/R injury.

**Conclusions and implications:**

These data provide the first evidence for cardioprotective activity of galanin G1 against myocardial I/R injury. Therefore, peptide G1 may represent a promising treatment strategy for ischemic heart disease.

## INTRODUCTION

Galanin is a highly conserved 29-(30 in human) amino acid neuropeptide with multiple biological functions. Galanin modulates the release and secretion of many neurotransmitters and hormones in the central nervous system and periphery, such as acetylcholine, gastrin, insulin, dopamine, somatotropin and prolactin. Central administration of galanin stimulates feeding behavior and energy balance that impact body weight regulation [1]. Galanin is involved in central cardiovascular regulation, which affects the blood pressure and the heart rate [2]. Administration of galanin into the rostral ventrolateral medulla produces a weak hypotension and tachycardia effect by reducing the sympathetic vasomotor tone in rats [3], which may be blocked by the intracerebroventricular injection of galanin antagonist M40 [4]. Recent studies suggest that galanin and its fragment play a major role in the regulation of metabolic homeostasis in cardiac muscle and galanin is an important hormone relative to diabetic heart [4]. The endogenous galanin, acting through its central receptor, has an important attribute to glucose transporter 4 (GLUT4) regulation, leading to enhanced insulin sensitivity and glucose uptake in cardiac muscle of type 2 diabetic rats [5]. In response to ischemia-reperfusion, galanin may stimulate sensory nerve regeneration and promote the regrowth of cardiac sensory nerves [6, 7]. Furthermore, galanin protects the heart muscle against hypoxia-induced contractile disturbances to improve inotropic action [8]. Although galanin and its fragments are involved in central cardiovascular regulation, the therapeutic potential of galanin-receptor ligands for coronary heart disease remains unexplored.

Galanin is involved in the regulation of physiological processes in peripheral organ systems via neuronal mechanisms and direct receptor mediated cellular effects. Currently three types of galanin receptors (GalR1, GalR2, and GalR3) have been identified by molecular cloning and characterized pharmacologically in various species [9]. They are members of the G-protein coupled receptor superfamily, but have differences in their functional coupling and signaling activities. All subtypes of galanin receptors are distributed in the hypothalamus, paraventricular nucleus, hippocampus, amygdale, peripheral nervous system and other tissues, including the heart [10]. The N-terminal end of galanin is highly conserved between different species, and the first 15 amino acid residues were found to be responsible for agonistic receptor binding [11]. The C-terminal region (amino acid residue 17-29) varies in most species and has a weak receptor affinity [11]. All three galanin receptor subtypes couple to G_i/o_ and inhibit adenylyl cyclase causing a decrease of the activity of the cAMP response element binding protein (pCREB). The activated pCREB may inhibit the Rab GTPase-activating protein (AS160), a substrate of Akt and GLUT4 translocation, thus promoting insulin resistance [11, 12]. GalR2 receptor signals through several classes of G-proteins and stimulates multiple intracellular pathways. Signaling via G_q_/11 activates phospholipase C (PLC) and protein kinase C (PKC) [13]. Activation of PLC leads to an increase in the hydrolysis of phosphatidylinositol (4,5) bis-phosphate (PI(4,5)P_2_) and promotes Ca^2+^ release from the endoplasmic reticulum [14], suggesting that galanin signaling via GalR2 receptor may modulate multiple cell death mechanisms in the failing heart. In spite of a variety of potential galanin receptor ligands developed to elucidate the specific roles of galaninergic system, very few agonists have high selectivity towards GalR2 receptor. One of them is a short N-terminal galanin fragment (2-11, G1) with no appreciable activation of GalR1 receptor [15]. The ability of this peptide to act as a non-GalR1 receptor agonist has provided evidence for the strong anti-kindling activities of G1. In fact, the action of G1 could be identified as anti-epileptogenic, as judged by the complete prevention of both full motor seizures and of a post-kindling increase of hippocampal excitability [16]. However, the peripheral action of this peptide remains poorly understood. No data regarding the role of G1 in cardiac cells and cardiovascular diseases is available so far.

In the present study we have evaluated the effects of G1 on myocardial I/R injury in various experimental models including cardiomyoblasts, perfused isolated heart and the heart *in situ*.

## RESULTS

### The effects of G1 on H9C2 cell survival in response to stress

**Figure 1:**
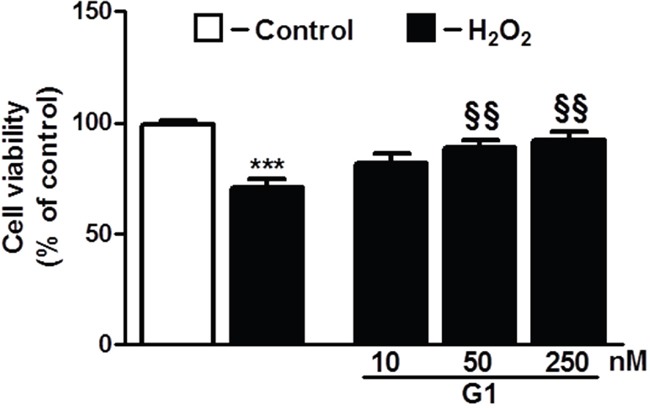
Dose-dependent effect of G1 on cell survival in response to oxidative stress Treatment of cardiomyoblasts with G1 prevents H_2_O_2_-induced decrease of cell viability in a dose-dependent manner. The H9C2 were pretreated with G1 (10, 50, 250 nM) for 20 min and then exposed to 400μM H_2_O_2_ for 4h. Values are the means ± SEM for three experiments. ***P < 0.001, vs control; *^§§^P* < 0.01, vs H_2_O_2_ treatment.

**Figure 2:**
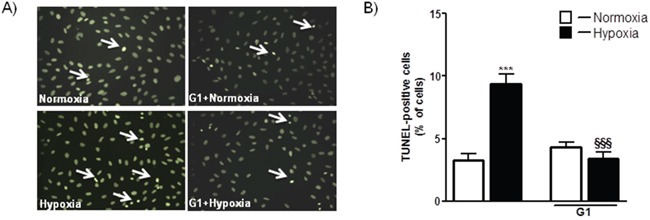
Effect of G1 on hypoxia-induced cell apoptosis **A**. Representative fluorescence images of H9C2 cells pretreated with 50 nM G1 for 20 min and then exposed to normoxia or hypoxia (1% O_2_) followed by reoxygenation. Apoptosis was measured by TUNEL assay in H9C2 cells after 16h of hypoxia followed by 4h of reoxygenation. **B**. Quantitative analysis of TUNEL-positive cells in H9C2 cells. Values are the means ± SEM from three experiments. ***P < 0.001, vs normoxia; *^§§§^P* < 0.001, vs hypoxia.

To determine whether G1 affects cell survival in response to oxidative stress, we examined the dose-dependent effects of the peptide on H_2_O_2_-induced loss of cardiomyoblast viability measured by ATP concentration. As shown in Figure 1, cell exposure to 400 μM H_2_O_2_ for 4 hours led to a significant reduction of the cell viability compared to control. Dose-response studies revealed that at the dose of 50 and 250 nM G1 was able to prevent H_2_O_2_-induced decrease of cell survival. Next, we examined by terminal deoxynucleotidyltransferase dUTP nick end labeling (TUNEL) assay whether G1 affects apoptotic cell death in response to hypoxic stress. Because 50 nM of G1 produced approximately a 20% increase in cardiomyoblast viability, we used this concentration in subsequent experiments. As shown in Figure 2, the exposure of H9C2 cells to hypoxia caused a significant increase in the number of TUNEL-positive cells as compared to normoxia. However, the treatment of cells with 50 nM G1 significantly reduced hypoxia-induced apoptosis (Figure 2A-2B).

### The effects of G1 on hypoxia-induced mitochondrial ROS production in H9C2 cells

**Figure 3:**
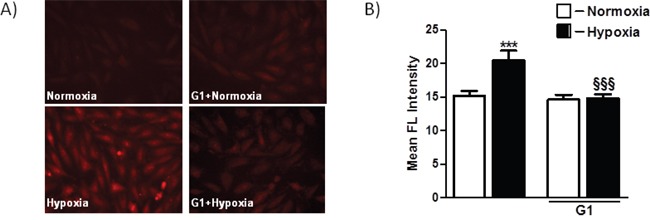
Effect of G1 on hypoxia-induced mitochondrial O_2_- production **A**. Representative fluorescence images of H9C2 cells pretreated with G1 peptide. Mitochondrial O_2_^−^ formation was assessed by MitoSOX Red in H9C2 cells exposed to 16h hypoxia followed by 4h of reoxygenation. **B**. Quantitative analysis of mitochondrial O_2_^−^ production in H9C2 cells exposed to normoxia or hypoxia-reoxygenation. Values are the means ± SEM from three experiments. ***P < 0.001 vs normoxia; *^§§§^P* < 0.001 vs hypoxia.

The excessive generation of ROS and impaired cellular metabolism are closely linked to cell death and myocardial damage [17]. To determine whether G1 could affect ROS generation in response to hypoxia, we examined the effects of G1 on mitochondrial superoxide (O_2_^−^) production using the MitoSOX Red fluorescent probe. As shown in Figure 3A-3B, cell exposure to hypoxic stress caused a significant increase in O_2_^−^ production as compared to normoxia. Importantly, treatment of H9C2 cells with 50 nM G1 markedly prevented hypoxia-induced O_2_^−^ formation (Figure 3A-3B).

### The cardioprotective potential of exogenous G1 in isolated rat hearts after I/R injury

**Figure 4:**
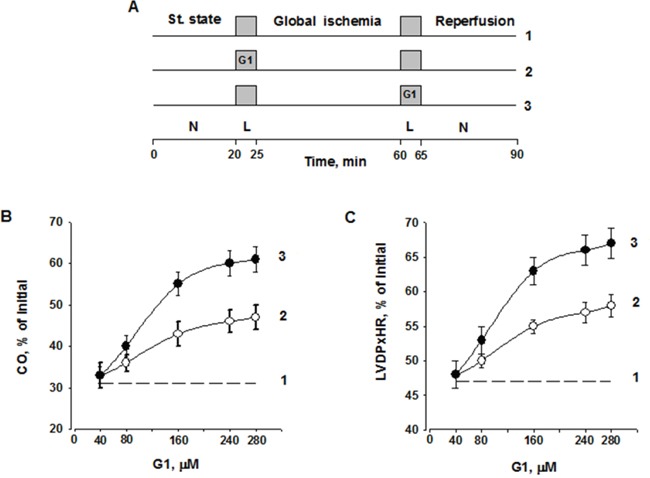
Dose-dependent effect of G1 infusion on functional recovery of isolated rat heart at the end of reperfusion **A**. Study design including three groups: 1 - Control; 2 - G1 infusion before ischemia; 3 - G1 infusion after ischemia; L - a 5-min Langendorff perfusion at a flow rate of 4 ml/min before or after ischemia; N, a working perfusion according to a method of Neely. **B**. Effects of peptide G1 on cardiac output (CO) recovery at the end of reperfusion. **C**. Effects of peptide G1 on the contractile function intensity index (left ventricular developed pressure (LVDP) × heart rate (HR)) recovery at the end of reperfusion. The values are expressed as means ± SEM from 8 experiments.

To study the functional role of galanin fragment in the failing heart, we evaluated the effects of G1 on the recovery of perfused hearts subjected to I/R injury (Figure 4A). Infusion of peptide G1 before global ischemia or at onset of reperfusion enhanced recovery of cardiac function during reperfusion compared with control. A dose-dependent effect of G1 on recovery of cardiac output (CO) by the end of reperfusion is shown on Figure 4B. The significant increase in CO recovery was observed after pre- or postischemic infusion of 80 μM G1 as compared with the control. The differences in CO recovery between the experimental and control groups became more pronounced with an increase in G1 concentration in Krebs-Henseleit bicarbonate buffer (KHB). The maximal response to G1 was observed at the concentration of 240 μM; at a higher concentration a dose-effect curve reached a plateau. Within the range of 80 - 280 μM, CO recovery was more effective when peptide infusion was performed after ischemia. A similar dose-dependent effects were obtained for recovery of the left ventricular (LV) developed pressure (LVDP) × heart rate (HR) product (Figure 4C).

Cardiac function indices were compared at the end of reperfusion for preischemic (G1-I) and postischemic (G1-R) infusion of the optimal G1 concentration (240 μM) in Table 1. In addition to recovery of CO, recovery of aortic output and stroke volume was also significantly higher in the G1-R group compared to the G1-I group. An augmented restoration of the LVDPxHR product in both G1 groups was due to better recovery of HR and LVDP comparing with control. A significant increase in LVDP was caused by a marked reduction of LV diastolic pressure during reperfusion. Both G1 groups exhibited an increase in coronary flow with concomitant reduction in coronary resistance in comparison with control. Data in Table 1 show that recovery of cardiac function was more effective after G1 administration at the onset of reperfusion.

**Table 1:** Effect of infusion of 240 μM G1 before (G1-I) and after global ischemia (G1-R) on recovery of isolated rat heats at the end of reperfusion

	Steady state	Control	G1-R	GI-I	Vehicle
**Coronary flow, ml/min**	17 ± 2	13 ± 1^a^	16 ± 1^b^	14 ± 1	13 ± 1 ^a^
**Perfusion pressure,mmHg**	62 ± 4	58 ± 1	60 ± 1	59 ± 1	58 ± 1
**Coronary resistance,mmHg/ml**	3.62 ± 0.03	4.46 ± 0.07^a^	3.95 ± 0.10 ^abc^	4.20 ± 0.13 ^a^	4.45 ± 0.13 ^a^
**LV systolic pressure,mmHg**	98 ± 3	69 ± 1^a^	85 ± 2 ^abc^	76 ± 3 ^ab^	69 ± 2 ^a^
**LV diastolic pressure,mmHg**	-3 ± 1	10 ± 1^a^	3 ± 1 ^abc^	6 ± 1 ^ab^	10 ± 1 ^ac^
**LV developed pressure,mm Hg**	101± 1	59 ± 2^a^	82 ± 3 ^ac^	70 ± 4 ^ab^	59 ± 3 ^a^
**Heart rate, beat/min**	301 ± 2	238 ± 3^a^	272 ± 5 ^abc^	254 ± 6 ^ab^	238 ± 5 ^a^
**LVDP x HR, mmHg/min**	30380 ± 373	14186 ± 525 ^a^	22318 ± 1270 ^abc^	17812 ± 1309 ^ab^	14052 ± 969 ^ac^
**Aortic output, ml/min**	26 ± 3	0 ± 1^a^	14 + 1 ^abc^	7 ± 1 ^ab^	0 ± 1 ^ac^
**Cardiac output, ml**	43 ± 2	13 ± 1^a^	29 ± 2 ^abc^	21 ± 2 ^ab^	13 ± 1 ^ac^
**Stroke volume, μl**	144 ± 1	54 ± 4^a^	106 ± 6 ^abc^	81 ± 8 ^ab^	53 ± 5 ^ac^

The hearts were perfused as indicated in Materials and methods. Data are the mean ± SEM for 10 experiments. ^a^ p<0.05 vs. steady state;^b^ p<0.05 vs. control and vehicle; ^c^ p<0.05 vs. G1-I.

**Figure 5:**
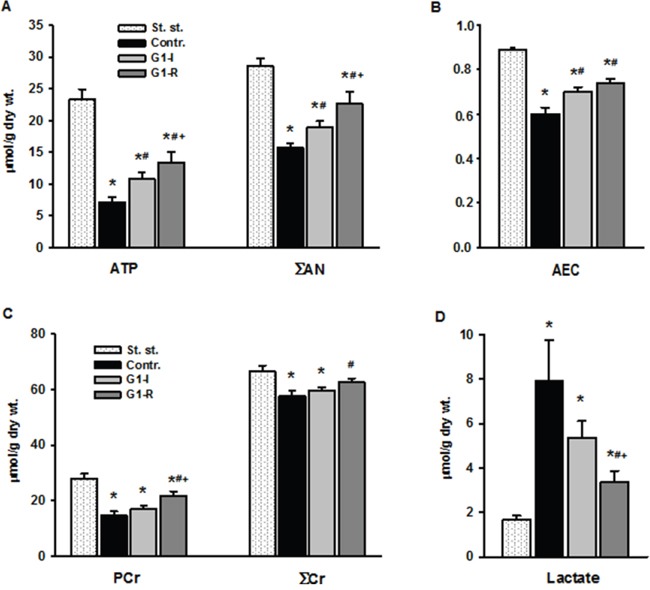
Effects of G1 infusion on metabolic state of isolated rat heart at the end of reperfusion St. - steady state; Contr. - control; G1-I - peptide infusion at the dose of 240 μM before ischemia; G1-R - peptide infusion at the dose of 240 μM after ischemia. **A**. Pre- or postischemic infusion of G1 improved recovery of myocardial ATP and adenine nucleotide pool (ΣAN) = (ATP+ADP+AMP) and **B**. adenylate energy charge (AEC) = (ATP+0.5ADP)/ΣAN) at the end of reperfusion. **C**. Infusion of G1 increased recovery of myocardial phosphocreatine (PCr), total creatine (ΣCr) = (PCr+Cr) and **D**. reduced myocardial lactate accumulation. Values are the means ± SEM from 8 experiments.*P < 0.05 vs. steady state; *^#^P* < 0.05 vs control, ***^+^****P* < 0.05 vs G1-I.

Myocardial energy status is a critical aspect of cardiac function. We next evaluated the effects of exogenous G1 on the energy metabolism in isolated hearts in response to I/R. The control group exhibited poor recovery of energy metabolism at the end of reperfusion. A dramatic decrease in myocardial ATP to 31% of the initial content was accompanied by a reduction of adenine nucleotide pool (ΣAN) and adenylate energy charge (AEC) by 45 and 33%, respectively, as compared to steady state values (Figure 5A-5B). Myocardial phosphocreatine (PCr) recovery was about 52% of the initial value, total creatine pool (ΣCr) was significantly reduced by 15%, while myocardial lactate content was almost 5 times higher than steady state value (Figure 5C-5D). Pre- or postischemic infusion of G1 significantly enhanced restoration of ATP, ΣAN and increased AEC in reperfused hearts compared with control. These effects were combined with a significant increase in PCr recovery, better preservation of ΣCr and a substantial reduction in myocardial lactate content. Recovery of metabolic state in the G1-R group was more effective than in the G1-I group. G1 infusion after ischemia improved preservation of ATP, ΣAN and PCr, and reduced lactate accumulation in myocardial tissue at the end of reperfusion in comparison with effects of preischemic peptide infusion. Thus, the experiments on perfused rat hearts clearly demonstrated metabolic and functional advantages of postischemic infusion with G1 over its administration prior to ischemia.

### The cardioprotective effects of exogenous G1 in anesthetized rats *in vivo*

In the steady state, there were no differences in the systolic arterial pressure (SAP) or HR between the groups (Table 2). Bolus injection of saline after the period of left anterior descending (LAD) coronary artery occlusion did not affect SAP and HR during reperfusion in control. Treatment with G1 at a dose of 0.4, 0.7 or 1.3 μmol/kg resulted in a fall in SAP (on average by 23±4% of the initial value at the second min of reperfusion). By the end of reperfusion, SAP recovered to near baseline (98±4%). A slight decrease in HR on average by 7±1% from baseline was observed in groups G1-0.4 and G1-0.7 at the first minute of reperfusion. It was accompanied by complete restoration of HR by the end of reperfusion. Similar hemodynamic changes were observed when G1 was administrated at a dose of 0.4 and 0.7 μmol/kg in sham-operated animals. In the G1-1.3 group, HR reduced to 73 ± 6% of the initial value at the first min of reperfusion and returned to baseline by the end of reperfusion. G1 administration at dose of 2.0 or 2.7 μmol/kg caused a pronounced bradycardia up to 200 and 100 beats/min, respectively and was accompanied by a sharp drop in SAP by 50 and 80%, respectively, followed by gain of bradycardia.

**Table 2:** Effects of G1 administration on systemic hemodynamic variables in anesthetized rats *in vivo*

Group	Steady state	LAD reperfusion	
		1-2 min	60 min
SAP, mm Hg			
**Control**	86 ± 3	85 ± 2	86 ± 3
**G1-0.4**	82 ± 2	63 ± 2 ^a b^	81 ± 4
**G1-0.7**	84 ± 2	64 ± 2 ^a b^	83 ± 2
**G1-1.3**	89 ± 3	69 ± 3 ^a b^	87 ± 2
HR, beats /min			
**Control**	334± 5	331 ± 4	332 ± 4
**G1-0.4**	338± 6	314 ± 7 ^a b^	337 ± 7
**G1-0.7**	329 ± 5	306 ± 6 ^a b^	330 ± 5
**G1-1.3**	332± 4	246 ± 4 ^a b c d^	327 ± 6

G1 was administrated by i.v. bolus injection at the onset of reperfusion at doses of 0.4; 0.7 or 1.3 μmol/kg (groups G1-0.4, G1-0.7 or G1-1.3, respectively). An equal volume of saline was injected in control. Values are expressed as mean ± SEM for 12 experiments. Significant difference (*P*<0.05) from: ^a^ steady state; ^b^ control; ^c^ G1-0.4; ^d^ G1-0.7.

**Figure 6:**
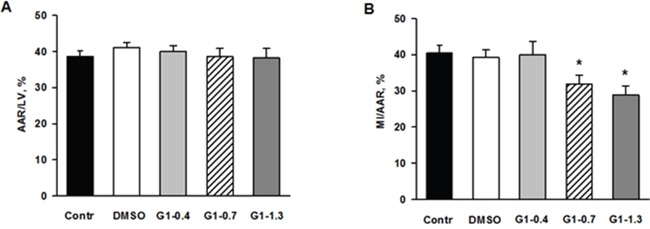
Effects of G1 on myocardial infarct size and area at risk in rats *in vivo* Dose-dependent effects of peptide G1 on **A**. area at risk (AAR) and **B**. myocardial infarct size. AAR was expressed as percentage of the left ventricular weight (AAR/LV, %) and myocardial infarct size (MI) was expressed as percentage of the AAR (MI/AAR, %). Control - i.v. bolus injection of 0.5 ml of saline; DMSO - i.v. bolus injection of 0.5% DMSO in saline; G1- i.v. bolus injection of peptide G1 at dose of 0.4, 0.7 or 1.3 μmol/kg. Values are the means ± SEM from 8 experiments.*P < 0.05 vs control.

**Figure 7:**
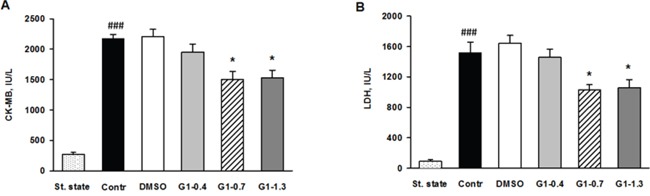
Effects of G1 on plasma level of necrosis markers in rats *in vivo* Dose-dependent effects of peptide G1 on **A**. activity of creatine kinase-MB (CK-MB) and **B**. lactate dehydrogenase (LDH) in blood plasma. St. state - steady state; Control - i.v. bolus injection of 0.5 ml of saline; DMSO - i.v. bolus injection of 0.5% DMSO in saline; G1- i.v. bolus injection of peptide G1 at dose of 0.4, 0.7 or 1.3 μmol/kg. Values are the means ± SEM from 8 experiments.*P < 0.05 vs Control; *^###^P* < 0.001 vs St. state.

We evaluated effects of G1 administration at doses of 0.4; 0.7 and 1.3 μmol/kg on myocardial infarct size and cell membrane damage. The percentage ratios of area at risk to LV weight (AAR/LV, %) were similar among G1-0.4, G1-0.7 and G1-1.3 groups and did not differ significantly from the values in control and the vehicle group (Figure 6A). Treatment with G1 at a dose of 0.4 μmol/kg or the vehicle alone did not affect the percentage ratio of myocardial infarction/area at risk (MI/AAR, %) compare with control and the vehicle group. Administration of G1 at a dose of 0.7 or 1.3 μmol/kg significantly reduced the percentage ratio of MI/AAR (on average by 25% compared with the value in control) thus indicating limitation of infarct size (Figure 6B). In addition, we analyzed the effects of G1 on plasma level of myocardial damage markers. In the steady state, plasma CK-MB and LDH activity of 270.1±24.2 and 90.3±15.5 IU/l were respectively observed (Figure 7A-7B). The activity of CK-MB and LDH in blood plasma increased by 8 and 16 times, respectively, by the end of reperfusion in the control animals. Administration of G1 at dose of 0.4 μmol/kg did not significantly affect the activity of both enzymes compared with control. Treatment with G1 at the dose of 0.7 or 1.3 μmol/kg reduced the CK-MB and LDH activity compared with the control group suggesting less damage to cell membranes.

## DISCUSSION

Galaninergic system has been implicated in diverse higher order physiological functions including cognition, feeding, nociception, mood regulation, and neuroendocrine modulation [18]. The peripheral functional properties of galanin and its receptors have not yet been fully elucidated. One major limitation to delineate the pathophysiological role of galaninergic system is the lack of receptor subtype specific ligands. Numerous studies within the galaninergic system have mainly been focusing on the GalR1 subtype, which therefore is the best-characterized among the three receptors. However, an intense interest has lately been addressed towards the other two receptors, GalR2 and GalR3. The increased attention for GalR2 coincides with the introduction of the GalR2 selective ligand G1, which has lower affinity for GalR3 but does not affect GalR1 [19, 20]. In the present study, we provide the first direct evidence that G1 has potent *in vitro* and *in vivo* cardioprotective activities against I/R-induced myocardial injury. Our data revealed that G1 prevents apoptotic cell death and mitochondrial ROS production under hypoxic stress in cardiomyoblasts. Furthermore, exogenous G1 attenuated cardiac damage, reduced infarct size and improved cardiac function in rat myocardial I/R injury suggesting the potential therapeutic value of G1 in heart diseases.

Compelling evidence has implicated a role for galanin and G1 in neuroprotection. For example, galanin knockout mice show a higher loss of pyramidal neurons in the hippocampus than wild-type mice after peripheral injection of kainic acid, and galanin or galanin (2-11) counteracts cell death induced by glutamate in hippocampal cultures [21, 22]. Our work demonstrates that exogenous G1 improves metabolic recovery of isolated rat heart subjected to I/R injury and cardiomyoblast survival after hypoxia and reoxygenation suggesting that galaninergic system may play an important role in metabolic remodeling of the heart in response to stress. Clinical studies and animal models demonstrate that abnormalities in cardiac energy metabolism are characteristic features of various heart diseases [23, 24]. The present results showing that the pre- or postischemic administrations of G1 improve recovery of myocardial energy status in response to I/R damage suggest that peptide G1 is an important regulator of cardiac metabolism in the failing myocardium. The ability of G1 to preserve energy state of postischemic myocardium may be related to enhanced uptake and utilization of glucose. At the metabolic level, administration of galanin antagonist M35 to diabetic rats reduces insulin sensitivity, decreases GLUT4 content [25], and reduces GLUT4 mRNA expression in the membrane of myocytes and adipocytes [26, 27]. Alterations in glucose utilization in the heart may be a metabolic indicator of progressing chronic heart failure. Indeed, in patients with diabetes and coronary artery disease, the loss of GLUT4 content in the heart takes place simultaneously with the development of severe heart failure and ischemic cardiomyopathy [28, 29]. Importantly, we show that improvement of myocardial energy metabolism by peptide G1 was associated with the preservation of contractile function in isolated rat heart after I/R injury. In addition, the exogenous administration of peptide G1 at the onset of reperfusion reduced infarct size and plasma levels of necrosis markers in rats subjected to I/R suggesting a beneficial role for peptide G1 in limiting myocardial ischemic injury. This is the first demonstration of a cardioprotective activity of peptide G1. Compared to the more selective synthetic GalR2 agonists M1153 and M1145 [12, 30], the main advantages of G1 are better solubility, the simplicity of chemical structure and the fact that this peptide is a fragment of natural galanin.

Recent findings indicate that changes in mitochondrial ROS production could be relevant in cardiovascular pathophysiology, as such alterations may have an impact on cellular fate decisions [24]. Excessive ROS generation in mitochondria may provoke a state of oxidative stress, associated with pathophysiological progression in heart ischemic diseases. The intracellular changes during ischemia, including accumulation of H^+^ and Ca^2+^ as well as the disruption of mitochondrial membrane potential, lead to the formation of ROS [31]. Furthermore, ROS accumulation directly activates the pathways of stress response, subsequently resulting in activation of apoptosis [32]. The primary ROS generated by mitochondria, as a result of mono-electronic reduction of O_2_, is superoxide anion O_2_^−^, the precursor of most ROS and a mediator in oxidative chain reactions [33]. In the present study, our results revealed that G1 prevents mitochondrial O_2_^−^ formation in living cardiomyoblasts and apoptosis in response to hypoxic stress. These findings suggest that G1 may control oxidative stress status and activation of apoptotic cell death pathways in cardiac cells. One of the principal results of our study is that G1-dependent increased cardiomyoblast survival in response to hypoxic stress was associated with reduced generation of mitochondrial O_2_^−^, a mediator in oxidative chain reactions. This association may be related to an increase in enzymatic antioxidant capacity induced by the peptide or to their direct antioxidant actions. Indeed, we have previously demonstrated that some endogenous peptides such as apelin, may exhibit powerful antioxidant properties [34, 35]. Further studies are required to define the potential mechanisms of how G1 inhibits mitochondrial ROS production and cell apoptosis and to identify the precise role of GalR2/GalR3 interactions in these processes.

In conclusion, our data provide the first evidence that the peptide G1 reduces I/R-associated cardiac dysfunction and myocardial damage. These beneficial effects are accompanied by substantial improvement of myocardial energy state and cell membrane integrity. In addition, we report that in cardiomyoblasts, G1 regulates mitochondrial ROS production and apoptosis in response to hypoxic stress. Taken together, these data collectively indicate that peptide G1 may be a promising new agent for cardioprotection against I/R injury.

## MATERIALS AND METHODS

### Galanin peptides

The peptide utilized in this study was rat galanin fragment G1 (Supplementary Table 1). Peptide G1 was synthesized by solid-phase method on Rink-amide-resin (Nova BioChem, Switzerland). The synthesis was carried out in automatic mode with Fmoc-(9-fluorenylmethoxycarbonyl)-technology on a peptide synthesizer Tribute-UV (Protein Technologies, Inc., USA). Fmoc amino acids (Nova BioChem, Switzerland) were coupled as 1-hydroxybenzotriazole esters. The peptide was cleaved from the resin using a solution of 95% trifluoroacetic acid, 2.5% triisopropylsilane and 2.5% H_2_O for 2 h. The crude product of solid-phase synthesis was purified by preparative reverse-phase HPLC with 98% homogeneity. The synthesized G1 has correct mass-spectrometric (m/z = 1137.3 [M+H]^+^) and ^1^H-NMR characteristics.

Analytical HPLC was performed on a Gilson (France) system using 4.6×250 mm Kromasil-100 5 μm C18 columns (Sweden). Preparative HPLC was performed on a Knauer (Germany) system using Eurosphere octadecylsilyl columns (20 × 250 mm, 10 μm) (Knauer, Germany). Acetonitrile (Panreac, Spain) was used for HPLC. The mass spectra were recorded on a VISION 2000 mass spectrometer (Termobioanalysis corp., Finnigan, United States) with matrix assisted laser desorbtion ionization time-of-flight (MALDI-TOF) method. ^1^H-NMR was performed on WH-500 Bruker 500 MHz (Germany) in DMSO-d6 at 300 K, peptide concentration was 2-3 mg/ml, chemical shifts in ^1^H-NMR spectra were measured relative to an internal standard tetramethylsilane.

### Reagents

Enzymes and chemicals for *in vivo* experiments were purchased from Sigma Chemical Co. (St Louis, MO USA). Solutions were prepared using deionized water (Millipore Corp. Bedford, MA, USA).

### Animals

Male Wistar rats weighing 300 to 340 g were housed in cages in groups of three, maintained at 20–30°C with a natural light-dark cycle. All animals had free access to standard pelleted diet (Aller Petfood, St. Petersburg, Russia) and tap water. The care and use of the animals were conducted in accordance with the European Convention for the Protection of Vertebrate Animals Used for Experimental and other Scientific Purposes (No 123 of 18 March 1986).

### Isolated perfused rat hearts

The isolated heart provides a highly reproducible preparation that can be studied in a time-dependent manner. It allows a broad spectrum of biochemical, physiological, morphological and pharmacological indices to be measured, permitting detailed analysis of ventricular mechanics, metabolism and coronary vascular responses. In principle, two different isolated heart models exist: 1) the isolated heart according to Langendorff (1895), in which hearts are supplied with coronary flow through retrograde perfusion and 2) the working, fluid-ejecting heart, in which hearts are perfused via the left atrium and eject fluid through the left ventricle into the aorta thus perfusing their own coronaries. The present study is designed with a combination of features from both the Langendorff (non-working) and the Neely (working) systems. The working heart performs pressure-volume work, an important distinction from its Langendorff counterpart, which performs energetically less demanding isovolumetric contractions.

Briefly, rats were heparinized (1600 IU/kg body weight, intraperitoneally (i.p.)) and anaesthetized with urethane (1.3 g/kg body weight, i.p.). Hearts were perfused with KHB containing (in mM): NaCl 118, KCl 4.7, CaCl_2_ 3.0, Na_2_EDTA 0.5, KH_2_PO_4_ 1.2, MgSO_4_ 1.2, NaHCO_3_ 25.0 and glucose 11.0. It was oxygenated with a mixture of 95%O_2_ and 5% CO_2_; pH was 7.4±0.1 at 37°C. KHB was passed through a 5 μm Millipore filter (Bedford, MA, USA) before use. A needle was inserted into the LV cavity to register LV pressure via a Gould Statham P50 transducer, SP 1405 monitor and a Gould Brush SP 2010 recorder (Gould, Oxnard, Ca, USA). The contractile function intensity index was calculated as the LVDP×HR, where LVDP is the difference between LV systolic and LV end-diastolic pressure. Cardiac pump function was assessed by CO, the sum of aortic output and coronary flow as previously described [34].

The steady state values of cardiac function were recorded after preliminary 20-min perfusion in working mode according to a modified method of Neely under constant left atrium pressure and aortic pressure of 20 and 100 cm H_2_O, respectively. After the steady state period, the control hearts were perfused in Langendorff mode for 5 min at a constant flow rate of 4 ml/min, and then they were subjected to 35-min normothermic global ischemia followed by 5-min Langendorff perfusion with subsequent 30-min working reperfusion by Neely method (Figure 4A).

In order to provide a successful G1 transport to cardiomyocyte membranes of ischemic heart we had to profoundly increase G1 concentration in KHB compared to cell experiments. Thus, in the G1-I group, 5-min Langendorff perfusion with KHB containing 40, 80, 160, 240 or 280 μM G1 was applied before global ischemia. Further procedure was the same as for the control group. The hearts of the G1-R group were perfused for 5 min with KHB containing 40, 80, 160, 240 or 280 μM G1 in Langendorff mode after global ischemia. Other experimental stages were the same as in control. The final concentration of dimethyl sulfoxide (DMSO) in KHB with peptide G1 was 0.2%. In a separate series of experiments, it was found that 5-min infusion of KHB containing 0.2% DMSO did not affect the recovery of cardiac function after ischemia.

### Analysis of metabolites

After preliminary working perfusion (steady state) and at the end of reperfusion, the hearts were freeze-clamped in liquid nitrogen for metabolite analysis. Frozen isolated perfused hearts were quickly homogenized in cooled 6% HClO_4_ (10 ml/g) using an Ultra-Turrax T-25 homogenizer (IKA-Labortechnik, Staufen, Germany), and the homogenates were centrifuged at 2800×g for 10 min at 4°C. The supernatants were then neutralized with 5M K_2_CO_3_ to pH 7.40, and the extracts were centrifuged after cooling to remove KClO_4_ precipitate. Tissue dry weights were determined by weighing a portion of the pellets after extraction with 6% HClO_4_ and drying overnight at 110°C. Concentrations of ATP, ADP, AMP, PCr, creatine (Cr) and lactate in neutralized tissue extracts were determined by enzymatic methods [36].

### *In vivo* rat model of I/R injury

Rats were anesthetized with 20% urethane (120 mg/kg, i.p.) and artificially ventilated with a KTR-5 animal respirator (Hugo Sacks Electronik) with a volume of 2–3 ml at a rate of 70–75 breaths/min. Further preparation of animals was performed as described earlier [37]. Arterial blood pressure was recorded with a pressure transducer (Statham p23Db, Oxnard, USA) using a polygraph Biograph-4 (St. Petersburg, Russia). The mean arterial pressure, heart rate and standard lead II ECG were recorded on a computer using a LabVIEW 7.1 data acquisition system (National Instruments, USA).

After 30-min stabilization of hemodynamic parameters (steady state), LAD coronary artery was occluded for 40 min to simulate regional ischemia; the duration of subsequent reperfusion was 1 h. In the experimental series, G1 was administrated by i.v. bolus injection at the onset of reperfusion at doses of 0.4; 0.7; 1.3; 2.0 or 2.7 μmol/kg (groups G1-0.4; G1-0.7; G1-1.3; G1-2.0 or G1-2.7, respectively). An equal volume of saline (0.5 ml) was injected in the control series of experiments. The influence of a vehicle, 0.5% DMSO, on myocardial infarct size was studied in a separate series of experiments. Additionally, effects of i.v. G1 administration on hemodynamic data were evaluated. At the end of experiments, LAD coronary artery was occluded and 2 ml of 2% Evans Blue (Sigma, USA) solution was injected through the jugular vein to distinguish the myocardial non-ischemic area from the AAR.

### Determination of myocardial infarct size

After staining with Evans Blue, the heart was excised and the LV was frozen. A frozen LV was transversely cut into 1.5 mm thick slices which were incubated in 0.1 M sodium phosphate buffer pH 7.4, containing 1% 2,3,5-triphenyl-tetrazolium chloride (TTC, Sigma, USA) 10 min at 37°C. The slices were fixed in 10% formalin for 5 min. Then they were placed between two transparent glasses and captured using a scanner at 600 d.p.i. resolution; the saved images were analyzed by computerized planimetry using Imagecal software. The slices were then weighed for determination of LV weight. The AAR was expressed as a percentage of LV weight; MI was expressed as a percentage of the AAR in each group.

### Determination of necrosis markers

At the end of the steady state and reperfusion, blood samples were collected for plasma separation. Plasma LDH activity was determined enzymatically with pyruvate as substrate by using standard kits from BioSystems S.A. (Barcelona, Spain). Plasma CK-MB activity was assessed by an immunoinhibition method using standard kits from BioSystems S.A. (Barcelona, Spain) from the rate of nicotinamide adenine dinucleotide phosphate formation by means of the hexokinase and glucose-6-phosphate dehydrogenase coupled reactions.

### Cell culture and treatments

Rat ventricular myocardial H9C2 cells were obtained from American Type Culture Collection (Manassas, VA, USA). H9C2 cells at passages 18 to 24 were seeded in 24 or 96-well cell culture plates with Dulbecco's Modified Eagle Medium (Invitrogen, Cergy-Pontoise, France) containing 10% Fetal Bovine Serum (Invitrogen, Cergy Pontoise, France), 100U mL^−1^ penicillin and 100μg mL^−1^ streptomycin at 37°C in a humidified atmosphere of 5% CO_2_ and were used at less than 80% of confluence.

### Measurement of mitochondrial O_2_^−^ production

The mitochondrial levels of ROS were determined in H9C2 cells subjected to hypoxia (1% O_2_, 5% CO_2_) for 16h followed by 4h of reoxygenation (95% O_2_, 5% CO_2_) using mitochondrial superoxide indicator (MitoSOX™ red, Life Technologies). Before hypoxia the H9C2 were pretreated with G1 analogue for 20 min. After reoxygenation, cells were washed once with phosphate-buffered saline (PBS) and incubated in 1μM MitoSOX red for 30 min at 37°C followed by three washes with PBS. The fluorescence was then measured at the excitation wavelength of 510 nM and emission wavelength of 580 nM.

### Evaluation of apoptosis

The apoptosis level was assessed using the TUNEL system according to manufacturer's instructions (Promega, Madison, WI, USA) as described previously [38]. TUNEL is a general method to detect nuclear DNA fragmentation during apoptosis. TUNEL technique relies on the use of endogenous enzymes that allow the incorporation of labeled nucleotides into the 3′-hydroxyl (3′OH) recessed termini of DNA breaks. The added value in this approach resides in the possibility of evaluating both morphological and staining features in the same sample.

### ATP measurement

ATP was measured with the CellTiter-Glo^®^ Luminescent Cell Viability Assay from Promega (Madison, WI). H9C2 cells were seeded at a density of 2 × 10^5^ cells/ml with 100μl per well in a white 96-well plate and allowed to grow for 24h. Before addition of 400μM of H_2_O_2_ for 4h, H9C2 cells were pretreated for 20min with G1 at the different doses (10, 50 and 250 nM). The cells were equilibrated at room temperature for 30 min and, followed by addition of the CellTiter-Glo^®^ reagents, per manufactured instructions. The luminescence was read after a 10 min incubation of the reagents on the INFINITE F500 on luminescence module (TECAN, Switzerland, Mennedorf) and expressed as mean percentage of control group.

### Statistical analysis

Data are presented as means ± SEM. Results were analyzed by one-way ANOVA followed by Bonferroni multiple range test post-hoc analysis for calculation differences between more than two groups. Comparisons between two groups involved use of the Student's unpaired t-test. All statistical analyses were performed using SigmaPlot version 12 (Systat Software Inc, San Jose, CA). A *p* < 0.05 was defined as significant.

## SUPPLEMENTARY MATERIALS TABLES


