# Supplementary material for: Myocardial protection from ischemia/reperfusion injury by exogenous galanin fragment
Source: Oncotarget. 2017 Feb 3;8(13):21241–52. doi: 10.18632/oncotarget.15071 (PMC5400580; doi:10.18632/oncotarget.15071)
Supplement: Supplementary file 1 [file oncotarget-08-21241-s001.pdf]

# Myocardial protection from ischemia/reperfusion injury by exogenous galanin fragment

## SUPPLEMENTARY TABLE

**Supplementary Table 1: Structure of Galanin and its analogues**

| Amino Acid Sequence | Denomination |
|---|---|
| **WTLNSAGYLL-$NH_2$** | **Galanin (2-11)** |
| GWTLNSAGYLLGPHAIDNHRSFSDKHGLT-$NH_2$ | Rat galanin |
| GWTLNSAGYLLGPPPALALA-$NH_2$ | M40 |
| GWTLNSAGYLLGPPPGFSPFR – $NH_2$ | M35 |
| RGRGNWTLNSAGYLLGPK(ε-NH-C(O)X)-$NH_2$ | M1153 |
| RGRGNWTLNSAGYLLGPVLPPPALALA-$NH_2$ | M1145 |
